# Socioeconomic position and incident mobility impairment in the Cardiovascular Health Study

**DOI:** 10.1186/1471-2318-7-11

**Published:** 2007-05-10

**Authors:** Cheryl K Nordstrom, Ana V Diez Roux, Richard Schulz, Mary N Haan, Sharon A Jackson, Jennifer L Balfour

**Affiliations:** 1 Wayne State University, Center for Health Research, Detroit, MI, USA; 2 University of Michigan, School of Public Health, Ann Arbor, MI, USA; 3 University of Pittsburgh, School of Medicine, Pittsburgh, PA, USA; 4 Centers for Disease Control and Prevention, Division for Heart Disease and Stroke Prevention, Atlanta, GA, USA

## Abstract

**Background:**

We investigated if personal socioeconomic position (SEP) factors and neighborhood characteristics were associated with incident mobility impairment in the elderly.

**Methods:**

We used data from the Cardiovascular Health Study, a longitudinal, population-based examination of coronary heart disease and stroke among persons aged 65 and older in the United States.

**Results:**

Among 3,684 persons without baseline mobility impairment, lower baseline SEP was associated with increased risk of incident mobility disability during the 10-year follow-up period, although the strengths of these associations varied by socioeconomic indicator and race/sex group.

**Conclusion:**

Among independent-living elderly, SEP affected development of mobility impairment into later life. Particular effort should be made to prevent or delay its onset among the elderly with low income, education, and/or who live in economically disadvantaged neighborhoods.

## Background

As life expectancy increases, the maintenance of independent living by the elderly is a growing public health concern in the United States. Among persons aged 65 years and older included in a US national prevalence survey of disability, 30% had difficulty with mobility, 13% had difficulties with basic Activities of Daily Living (ADL), comprised of eating, bathing, dressing, transferring, and toileting, and 20% reported difficulty with Instrumental Activities of Daily Living (IADL), comprised of getting around outside of home, taking care of money and bills, preparing meals, doing light housework, and using the telephone [1]. Understanding contributors to the development of physical functional impairment will foster interventions to prevent or delay onset of mobility limitations in the elderly with the potential added benefit of large-scale cost savings as the elderly are kept out of nursing homes and assisted-living facilities.

There is clear evidence that persons with lower socioeconomic position (SEP) experience higher morbidity and mortality rates for many illnesses compared with persons with higher SEP [2-6]. It is less clear whether these SEP disparities in health continue into old age [7-12] and whether SEP impacts later-life development of mobility problems among the elderly with no previous mobility limitations [13-15]. Although there has been much recent interest in neighborhood health effects [16], data relating neighborhood characteristics to physical functioning in the elderly remain limited [17,18].

Berkman and Gurland found strong cross-sectional associations between household income and functional impairment, as ascertained by the Activity Limitation Scale of the SHORT-CARE, among adults aged 65+ in the Growing Old in New York City Study [7]. For each $10,000 increase in income, there was a one-point decrease in the ALS score (range 0–27). They also found an inverse association with education adjusted for demographic factors and income, such that the ALS score decreased by 1 point for every 6.7 years of education attained. Another study reported similar cross-sectional associations between income, but not education, and physical functioning among the elderly in the United States; however the income differential was noted only among those 60–65 years old [19]. A handful of studies have begun to examine the relationship between neighborhood characteristics and physical functioning. Higher neighborhood SEP may positively influence perceived safety and walkability, leading to more physical activity and promoting continued independence for older persons. Robert found that the percentage of households receiving public assistance and the percentage of families with income $30,000, but not percent of adult unemployment, were associated with increased odds of functional limitation [18]. These associations remained when all three community-level variables were entered into the regression model, controlling for age, sex, and race. However, none of the neighborhood factors was independently related to functional limitation when individual-level (income and education) and family-level (dollar value of assets) SEP indicators were taken into account.

The cross-sectional studies reviewed above are limited in their ability to draw conclusions regarding causal effects of socioeconomic position on the development of disability. Information regarding the relationship between socioeconomic position and incident disability remains limited and conflicting. For example, data from the Alameda County Study and the Americans' Changing Lives cohorts show associations between SEP and changes in physical functioning over time among adults with a wide range of functioning [13,20], as does data from the Health ABC Study [21], but other studies do not support these findings [22,23].

Very few studies have examined the relationship between neighborhood characteristics and incident disability. Data from the Alameda County Study showed that among persons without severe limitation at baseline, the odds of physical function loss over a 30-year follow-up was 50% higher among persons living in neighborhoods with one "problem" (i.e., traffic, noise, crime, trash and litter, lighting, and public transportation) and nearly 2.5 times higher among those in areas with more than one "problem," compared with persons living in neighborhoods with no such problems [17]. The gap between low and high categories of problems remained significant after adjustment for personal SEP (income and education), social connectedness, health status, and health behaviors.

Using data from a large, population-based longitudinal study of adults 65 years of age and older in the United States, we investigated if personal SEP and neighborhood characteristics are related to incident mobility impairment in the elderly. Mobility was selected because it represents an important domain of physical functioning essential to continued independent living among older adults [14]. Further, mobility limitations are the most prevalent indicator of disability in the United States [1] and are the most likely impairments to be impacted by neighborhood environment (i.e. walkability), as opposed to other assessments of functional limitations in the elderly, such as the Activities of Daily Living scale [24], which focus on limitations occurring largely within the context of the home [17].

## Methods

### Study population and study variables

The Cardiovascular Health Study (CHS) is a population-based longitudinal study of coronary heart disease and stroke in US adults aged 65 years and older [25]. In 1989–1990, 5,201 men and women were recruited from Medicare eligibility lists in four communities: Forsyth County, North Carolina; Sacramento County, California; Washington County, Maryland; and Pittsburgh, Pennsylvania. Due to small numbers of Blacks in the original cohort, in 1992–1993 an additional 687 Black participants from three of these four geographic locations (excluding Washington County, MD) were recruited into the study. Follow-up visits were conducted yearly through 1998–99, for a maximum of 10 follow-up visits over this time period (7 for those recruited later). Overall, 61% of eligible persons agreed to participate in the Cardiovascular Health Study [26].

Physical functioning was assessed using self-report at the baseline visit and at yearly follow-up visits. Questions were based on two modified items from the Rosow-Breslau Functional Health Scale [27] and categorized similarly to work of Guralnik et al [14] such that mobility impairment was considered present if the participant reported difficulty either 1) walking a half-mile or 2) walking up ten steps. These two questions have also been used in similar studies [21].

Personal SEP information was obtained from the baseline interview. Educational attainment was reported to the interviewer and was included in the analyses as an ordinal categorical variable based on four education groups (incomplete high school, complete high school or GED, some college or vocational training, 4 years of college or more). Personal income was defined as total family income before taxes from all sources in the past 12 months and was selected from a response card as one of the following: under $5,000; $5,000 to $7,999; $8,000 to $11,999; $12000 to $15999; $16,000 to $24,999; $25,000 to $34,999; $35,000 to $49,999; over $50,000. For these analyses, income was modelled as an ordinal categorical variable based on four income groups (< $12, 000, $12,000 to $24,999, $25,000 to $34,999, ≥ $35,000).

Neighborhood socioeconomic characteristics were summarized in a neighborhood score based on census-defined block-groups. The score was constructed by combining six variables derived from 1990 U.S. Census data for block-groups based on the home address the participant reported at the baseline examination [28]. Variables included in the summary score represent neighborhood income and wealth (log of the median household income, log of the median value of housing units, and the percentage of households receiving interest income); neighborhood education (the percentage of adults 25 years of age or older who had completed high school and the percentage of adults 25 years of age or older who had completed college); and neighborhood occupation (the percentage of employed persons 16 years of age or older in executive, managerial, or professional specialty occupations). For each variable, a z-score, reflecting the deviation of the value from the mean across all block-groups in the sample was calculated and the sum of these standardized values was deemed the neighborhood score. Neighborhood score in this sample ranged from -10.7 to 16.7, with higher scores representing greater neighborhood affluence. Distributions of neighborhood score were markedly different for Whites and Blacks (mean ± SD Whites 3.3 ± 4.6, mean ± SD Blacks -2.6 ± 4.2). Although it precludes direct comparison of results for Blacks and Whites, these disparate distributions necessitated that neighborhood score be based on race-specific quartiles of the score, modelled as an ordinal categorical variable. There were a total of 548 block groups, with a median of 4 participants per block group and 37% of block groups had more than 5 participants

In addition to examining the individual effects of education, income, and neighborhood score, a combined approach using a summary SEP score was constructed as a sum of the ordinal rankings for income category (1 to 4), education category (1 to 4), and block-group quartile (1 to 4). Summary SEP could therefore range from a minimum of 3 (lowest SEP) to a maximum of 12 (highest SEP).

Of 5,888 participants at baseline, 5,127 could be matched to block-groups with available census data. After excluding those who were neither Black nor White, or who were missing data on educational attainment or mobility, there were 4,884 participants. Including only persons without mobility impairment at baseline left 3,684 persons for these analyses (this excluded 607 White females, 292 White males, 223 Black females, and 78 Black males).

### Statistical analyses

Means and percentage distributions of SEP indicators and mobility impairment were calculated for each race/gender group. Time-to-event analysis was used to examine associations of personal and neighborhood SEP with incident mobility impairment and was conducted using age-adjusted proportional hazards regression models fit with robust variance estimates to account for potential effects of neighborhood clustering [29]. Persons with baseline mobility impairment were excluded from these incidence analyses. Time-to-event was defined as time from baseline to the midpoint between the visit at which mobility impairment was first identified and the prior visit, regardless of the impairment status at later visits. Those lost to follow-up were censored at the time of the last recorded visit. Persons with missing data on intermediate visits were censored at the time of the last consecutive visit.

Analyses were conducted using the SAS statistical software package, version 8.2 (Cary, NC) [30]. All participants gave written informed consent and all study protocols were approved by the Institutional Review Boards of participating institutions.

## Results

Among Whites, approximately 50% had information on physical functioning for all 10 visits, 25% had information for 7–9 visits, 16% had information for 4–6 visits and 11% had information for 3 visits or less. Among Blacks, approximately 38% had information on physical functioning for all 7 possible visits, 27% had information for 4–6 visits, and 19% had information for 3 visits or less. Fifty-eight percent of the first cohort of Blacks (16% of the total Black sample), with baseline visits during the same time period as Whites, contributed data from 8–10 visits. Among those without baseline mobility impairment, the incidence rate of mobility impairment was 11 cases per 100 person-years for White females, 9 per 100 person-years for White males, 15 per 100 person-years for Black females, and 11 per 100 person-years for Black males.

CHS study participants were drawn from four heterogeneous sites. Adjustment for CHS study site did not affect the magnitudes of the associations between any SEP indicator and incident mobility impairment in Whites or Blacks. We also tested for interactions between CHS study site and the various SEP indicators as related to mobility. The addition of site by SEP interactions significantly improved the fit of the model in only 3 of the 12 models tested: there was a stronger inverse association of income with incident mobility impairment among both Black and White men from North Carolina compared with Pennsylvania; the association of education with mobility impairment was weaker among White men from California compared with Pennsylvania (data not shown).

At baseline, there were fewer White women and men in the lowest income group (< $12,000 per year; 10% and 3%, respectively) than Black women and men (40% and 15%, respectively). Whites were concentrated in the "complete high school" category (44% of women and 33% of men), while the majority of Blacks were clustered in the "incomplete high school" category (38% of women and 38% of men). Blacks also tended to live in more economically disadvantaged neighborhoods compared to Whites. (Table 1)

Among persons without mobility impairment at baseline, income, education, and neighborhood score at baseline were inversely associated with increased risk of incident mobility impairment during the follow-up period among Whites, although the trend test for education was only marginally significant among White men (Table 2, Model 1). Among Whites, hazard ratios comparing the lowest vs. the highest socioeconomic category ranged from 2.06 (CL 1.39–3.05) for income among men to 1.23 (CL 1.03–1.44) for neighborhood score in women. Simultaneous adjustment for all socioeconomic indicators weakened the trends observed. Patterns remained in the expected directions but only trends for education in White women and income in White men remained statistically significant (Table 2, Model 2).

Findings for Black women were generally similar to those reported for Whites: lower income and education at baseline were associated with increased hazards of mobility impairment. Hazard ratios comparing the lowest to the highest category ranged from 3.55 (CL 1.68 to 7.47) for income to 1.30 (hazard ratio 0.84 to 2.02) for neighborhood score. In Black men, associations were in the same direction as in Black women but associations were substantially weaker and none of the estimates differed significantly from 1.0 at the 0.05 level. As in the case of Whites, simultaneous adjustment for all socioeconomic indicators weakened the associations observed and the trend remained statistically significant only for income in Black women. (Table 2, Model 2). A higher summary SEP score was significantly associated with lower hazards of developing mobility impairment in all race/gender groups, except among Black men where the 95% confidence limits include 1.0.

## Discussion

In this elderly cohort, the development of mobility impairment was positively associated with low income, low education, and living in a disadvantaged neighborhood. A summary SEP score that combined income, education, and neighborhood SEP was also related to incident mobility impairment. These associations were weaker and were not statistically significant in Black men. Our results regarding socioeconomic differentials are consistent with data from other studies, including the Americans' Changing Lives study which showed that higher income and education were protective against incident activity limitation [20]. However, only 25% of the ACL study sample was over age 60, while our sample was entirely aged 65 and older at baseline, demonstrating that the effects of SEP on mobility continue into older age. Our findings also echo previous investigations relating functional limitations to poor neighborhood conditions [18], although only among White participants.

We also investigated associations of income, education and neighborhood score with mobility impairment before and after adjustment for each other. Spearman correlation coefficients between the three ordinal measures used in the analyses were in the 0.22 to 0.50 range. In general the associations with each socioeconomic indicator were weakened and often became non-statistically significant after adjustment for each other. Limited power may have hampered our ability to detect "independent" effects of income, education and neighborhoods on incident mobility. Moreover, because these indicators are obviously tightly linked in the real world, associations with the combined SEP score may be better indicators of the overall gradient than the artificial, "independent" effects of the three measures.

The use of income as a measure of SEP may be less relevant in the elderly, when people are more apt to have limited earnings that may not reflect the level of earnings prior to retirement. More subjective measures, such as perceived income adequacy, may be better suited for use among older persons, and has been associated with development of disability (e.g., ADL) among those aged 75 and older [31]. Additionally, number of persons supported by the reported income would have provided a more complete measure of income. Unfortunately, measures of income adequacy, wealth, or number of persons supported by a given income were not available in our study. Our incomplete measure of income for an elderly population limits our ability to assess the magnitude or direction of bias it introduces. However, it is striking that even the limited measure of income that we had was strongly associated with mobility impairment in our analyses.

Self-report of mobility may have resulted in some misclassification of subjects and could show different SEP-patterning than more objective measures of mobility. A report based on objective, performance-based measures showed cross-sectional associations between income/education and performance-based physical capacity, but no relationship between SEP and change in capacity over time [32]. However, it is unclear how our findings may have been affected by using self-reported rather than objective measures of mobility. Further examination of the issue is warranted.

While inverse associations between SEP and incident impairment were evident among persons without impairment at baseline, our sample is necessarily limited to persons who had reached older ages in a relatively healthy state (i.e., survivors). If survivorship is associated with both socioeconomic factors and mobility impairment, our results could underestimate the relationship between social factors and the development of mobility over time. This could also explain the absence of socioeconomic differentials in Black men among whom attrition due to death or loss to follow-up were greatest. Findings for Black participants were further limited by the small sample size and shorter follow-up for that group (many of whom were recruited into the study at a later stage). Because of the disparities in neighborhood SEP distribution, we calculated quartiles of the block group score separately for Blacks and Whites, resulting in categories that are very different in Whites and Blacks and thus racial comparisons of the effect of neighborhood SEP (and also the summary SEP measure) on mobility cannot be made. Limited sample size did not allow estimation of associations for Whites living in very low SEP areas or Blacks living in very high SEP areas.

Another factor that could potentially impact our findings is differential attrition according to SEP. While we did find differences based on income, losses were similar in the both the low and high income groups, with both being greater than for those in the middle income range (data not shown). There were no differences in attrition based on education or neighborhood score. Additionally, those with prevalent mobility impairment at baseline (who were excluded from these analyses) were significantly less likely to be in the highest quantile of income, education, or neighborhood score (all p < 0.001), which could underestimate the noted association between SEP and incident impairment.

In looking at neighborhood effects on mobility, we are limited by the characterization of neighborhoods based on aggregate socioeconomic characteristics, which may not adequately capture the specific features of neighborhoods that are directly relevant to changes in functional status over time, such as perceptions of safety or walkability that may limit leaving the home and reduce opportunities for mobility. Further, our neighborhood measures were only from the baseline visit, though the cohort was quite stable over follow-up, making it unlikely that participants' neighborhood SEP changed significantly over time. Information on block-group of residence for the latest updated address available in 1998 was obtained for 4,665 CHS participants (95% of the sample with complete baseline information). Of these, 69% lived in the same block-groups at both times, although those without mobility impairment at baseline were more likely to live in the same place than those with baseline mobility impairment (71% versus 65%, χ^2 ^= 13.5, p = 0.0002). In addition, having mobility impairment at any time during the follow-up was not associated with moving to a neighborhood with a lower score.

## Conclusion

Our study shows longitudinal associations between SEP and incident mobility impairment in a diverse elderly population. Further investigations of factors associated with the development of impairment at earlier ages and over the lifecourse, including those related to neighborhood of residence, are needed to better understand the reasons for the continued socioeconomic gradient at older ages. Studies that specifically examine the mechanisms through which socioeconomic factors affect functional status may contribute to the development of more effective prevention strategies. For example, obesity and other comorbidities that disproportionately impact those with low SEP and also impact mobility may mediate the association and offer at least a partial explanation, although findings from the GLOBE study indicate that neither psychosocial and behavioral factors nor disease severity and comorbidities impacted the association among persons with chronic conditions [33]. Biomedical factors, including heart disease, hypertension, and inflammatory markers have also been partially implicated in the association between low SEP and functional decline in the Health ABC Study, and thus may provide further rationalization for efforts to reduce these factors, especially among those with low income and/or education [21]. Other socioeconomic factors, such as access to health care, which is often implicated in the association between poverty and poor health, may prevent early diagnosis and treatment of injuries that progress to loss of mobility. Other studies should test whether neighborhood factors are related to indicators of functional impairment other than mobility, which can then inform intervention design.

Since our findings show that SEP continues to impact the development of functional limitations in the elderly, health promotion activities aimed at prolonging independent living by maintaining mobility should target individuals with low education or income and those living in disadvantaged neighborhoods. Identifying specific mechanisms responsible for this socioeconomic patterning will allow the development of more specific prevention strategies aimed at minimizing or compensating for decreased mobility (e.g. access to assistance with chores and errands). Community development efforts that improve walkability and safety may also encourage walking and perhaps decrease mobility impairment.

## Competing interests

The author(s) declare that they have no competing interests.

## Authors' contributions

CKN led the writing of the paper and conducted the analyses; AVDR contributed to the design of the analyses and the writing of the paper, and supervised the work; RS, MNH, SAJ, JLB all critically reviewed several drafts of the manuscript. All authors read and approved the final manuscript.

## Pre-publication history

The pre-publication history for this paper can be accessed here:



## Figures and Tables

**Table 1:** Baseline personal socioeconomic and neighborhood characteristics, and incident mobility impairment among 3,684 participants included in the analyses

	**Full Sample**	**Whites**		**Blacks**	
		**Women**	**Men**	**Women**	**Men**
	(n = 3,684)	(n = 1,702)	(n = 1,452)	(n = 296)	(n = 234)

**Age in years (SD)**	72 (5)	72 (5)	73 (6)	72 (5)	72 (5)
					
**Income (%)**					
< $12,000	10	10	3	40	15
$12,000–$24,999	25	27	21	25	33
$25,000–$34,999	33	32	29	19	30
≥ $35,000	25	23	32	9	16
Unknown	7	8	5	7	6
					
**Education (%)**					
Incomplete High School	24	21	23	38	38
Complete High School or GED	37	44	33	29	26
1–3 years College or Vocational	15	16	15	14	15
4 Years College or More	23	19	30	19	21
					
**Neighborhood score**					
Median (25^th^,75^th^)	2.8 (-0.9, 6.2)	3.2 (0.0, 6.7)	3.3 (0.0, 6.8)	-3.5 (-5.1, 0.3)	-3.2 (-5.4, 0.85)
					
**Incidence of mobility impairment (cases/100 person-years)**	10	11	9	15	11

**Table 2:** Hazards ratios (with 95% confidence intervals) of incident mobility impairment among those without impairment at baseline, by socioeconomic indicators

	**Whites**				**Blacks**			
	Women (n = 1,702)		Men (n = 1,452)		Women (n = 296)		Men (n = 234)	

	Model 1	Model 2	Model 1	Model 2	Model 1	Model 2	Model 1	Model 2

Neighborhood Score*								
1^st ^quartile	1.23 (1.03,1.44)	1.12 (0.93, 1.35)	1.30 (1.05, 1.62)	1.15 (0.89, 1.48)	1.30 (0.84, 2.02)	1.16 (0.75, 1.80)	1.14 (0.65, 1.97)	1.01 (0.55, 1.86)
2^nd ^quartile	1.19 (1.00, 1.42)	1.12 (0.94, 1.34)	1.28 (1.04, 1.57)	1.19 (0.95, 1.50)	1.09 (0.68, 1.76)	0.97 (0.60, 1.57)	1.05 (0.59, 1.90)	1.01 (0.55, 1.83)
3^rd ^quartile	1.04 (0.88, 1.23)	1.01 (0.85, 1.21)	1.21 (0.98, 1.49)	1.18 (0.95, 1.47)	1.38 (0.88, 2.14)	1.34 (0.87, 2.07)	0.94 (0.54, 1.66)	0.96 (0.54, 1.71)
4^th ^quartile	REF	REF	REF	REF	REF	REF	REF	REF
P Trend	0.009	0.21	0.01	0.38	0.41	0.38	0.63	0.80
								
**Income**								
< $12,000	1.29 (1.01, 1.64)	1.12 (0.87, 1.45)	2.06 (1.39, 3.05)	1.92 (1.27, 2.91)	3.55 (1.68, 7.47)	3.01 (1.28, 7.10)	1.15 (0.53, 2.49)	1.10 (0.42, 2.85)
$12,000–$24,999	1.20 (1.01, 1.43)	1.07 (0.88, 1.29)	1.32 (1.06, 1.64)	1.23 (0.95, 1.60)	2.85 (1.34, 6.10)	2.61 (1.14, 5.89)	1.28 (0.70, 2.35)	1.22 (0.56, 2.64)
$25,000–$34,999	1.08 (0.90, 1.29)	1.01 (0.84, 1.21)	1.11 (0.93, 1.33)	1.06 (0.87, 1.30)	2.31 (1.02, 5.25)	2.25 (0.96, 5.28)	0.85 (0.45, 1.61)	0.85 (0.42, 1.74)
≥ $35,000	REF	REF	REF	REF	REF	REF	REF	REF
P Trend	0.01	0.38	0.0004	0.01	0.0002	0.004	0.29	0.40
								
**Education**								
Incomplete High School	1.43 (1.17, 1.74)	1.29 (1.04, 1.60)	1.25 (1.02, 1.55)	1.07 (0.85, 1.35)	1.91 (1.18, 3.08)	1.28 (0.74, 2.21)	1.29 (0.75, 2.21)	1.12 (0.57, 2.19)
Complete High School or GED	1.28 (1.08, 1.52)	1.22 (1.02, 1.46)	1.06 (0.87, 1.27)	0.94 (0.76, 1.15)	1.35 (0.84, 2.16)	1.01 (0.61, 1.65)	0.98 (0.51, 1.89)	0.94 (0.42, 2.09)
1–3 yrs College or Vocational	1.31 (1.08, 1.60)	1.29 (1.05, 1.57)	1.07 (0.84, 1.36)	1.00 (0.78, 1.29)	1.10 (0.59, 2.04)	0.87 (0.48, 1.59)	1.15 (0.57, 2.33)	1.06 (0.46, 2.42)
4 Years College or More	REF	REF	REF	REF	REF	REF	REF	REF
P Trend	0.001	0.03	0.06	0.64	0.006	0.85	0.43	0.98
								
**Summary SEP score (per unit change)**	0.95 (0.93, 0.98)	n/a	0.95 (0.92, 0.98)	n/a	0.89 (0.82, 0.96)	n/a	0.96 (0.88, 1.05)	n/a
P Trend	0.0004	n/a	0.0009	n/a	0.002	n/a	0.37	n/a

*race-specific quartiles

**Model 1 adjusted for age; Model 2 adjusted for age plus all other SEP indicators
